# Assessment of extremity dose for medical staff involved in positron emission tomography/computed tomography imaging: Retrospective study

**DOI:** 10.1097/MD.0000000000035501

**Published:** 2023-10-27

**Authors:** Rabiye Uslu Erdemir, Mohamed Mahmoud Abuzaid, Baris Cavli, Huseyin Ozan Tekin, Wiam Elshami

**Affiliations:** a Zonguldak Bülent Ecevit University of Medicine, Department of Nuclear Medicine, Zonguldak, Turkey; b Medical Diagnostic Imaging Department, College of Health Sciences, University of Sharjah, Sharjah, United Arab Emirates; c Research institute for medical and health sciences; d Affidea, Istanbul, Turkey; e Istinye University, Faculty of Engineering and Natural Sciences, Computer Engineering Department, 34396, Istanbul, Turkey

**Keywords:** extremity dose, medical workers, nuclear medicine, occupational dose, OSL, PET CT, radiation, radiation protection, radiation workers

## Abstract

There has been an increase in positron emission tomography (PET)/computed tomography (CT) imaging procedures, and medical workers involved in PET/CT are at increased risk of occupational exposure. Data on extremity dose exposure are limited globally. The current study aimed to evaluate the occupational radiation dose for extremities for medical workers (nurses, radiographers/radiologic technologists, and nuclear medicine physicians) working in PET/CT scanners at 5 large hospitals in Turkey. Optically stimulated luminescence (OSL) and Thermoluminescent dosimeter (TLD) ring dosimeters were used to measure equivalent dose values. Hospitals 1, 2, and 5 used OSL, and 3 and 4 used TLD. A total of 502 readings were obtained from 55 workers. In millisievert (mSv), the average annual effective dose for all workers was 14.5 ± 17.7 (0.2–157.2). A radiography technologist received a maximum dose of 157.21. Nurses received the highest average annual effective dose (15.2 ± 19.46) (0.32–65.58), followed by radiography technologists (14.7 ± 18.03) (0.4–157.2), and nuclear medicine physicians demonstrated the least dose (8.6 ± 10.5) (1.2–24.4). The results show that the extremity dose is well below the annual dose limit of 500 mSv. However, there is a wide variation in dose among the workers, underlining a need for careful assessment of working conditions to ensure safe practices for all workers.

## 1. Introduction

Positron emission tomography (PET) and computed tomography (CT) are noninvasive imaging modalities. While CT offers morphological images of the organ, PET provides a semiquantitative assessment of the function. The combination of anatomy and function provides valuable diagnostic information about internal organs. Therefore, PET/CT procedures have increased *substantially* in recent years.^[1]^ PET uses the radiopharmaceutical Fluorine-18 18F-fluorodeoxyglucose to trace glucose metabolism. It has a half-life of 109 minutes and a positron emission of 630 keV. Fluorine-18 18F-fluorodeoxyglucose PET/CT is a sensitive primary staging tool guiding patient care during cancer assessment.^[2]^

The PET/CT involves performing many tasks that can contribute considerably to increasing the occupational radiation dose absorbed by the extremity of medical staff, including the preparation, dispensing and patient administration of radiopharmaceuticals.^[3]^ Many studies document that the annual occupational radiation dose for medical workers in PET/CT is within the accepted dose limit, below 20 millisievert (mSv). In addition, studies have found that handling radioactive materials contributes to 60% of the occupational dose.^[4]^

According to the International Commission on Radiation Units and Measurements, the radiation dose limit to the extremity is 500 mSv per year. Extremity dose measurement uses the personal dose equivalent Hp (0.07). However, the increase in PET/CT examinations combined with the developments in radiopharmaceuticals raised concerns about future radiation exposure for medical workers.^[3]^

Therefore, it is essential to analyze and estimate radiation doses to medical staff working in PET/CT and their risk of exposure in the years to come, taking into account current data on the number and type of PET/CT procedures used, the number of employees involved, workload allocation among staff members, and radiation protection measures implemented.

Occupational radiation dose depends on the worker skill in implementing the protection principles, including time, distance, and shielding. Therefore, reducing radiation doses to workers in a PET/CT facility is a complicated issue, and more effort is required to implement the as low as reasonably achievable principles and International Commission on Radiological Protection recommendation.^[1]^ The average annual dose limits for the effective dose, whole body dose Hp(10) and the equivalent dose for extremities Hp(0.07) are 20 mSv and 500 mSv, respectively.^[5]^

The present study aimed to analyze the occupational radiation exposure to the skin of medical workers in PET/CT to estimate the radiation exposure level and determine the differences in the radiation dose level between workers.

## 2. Methods

**Figure 1.**
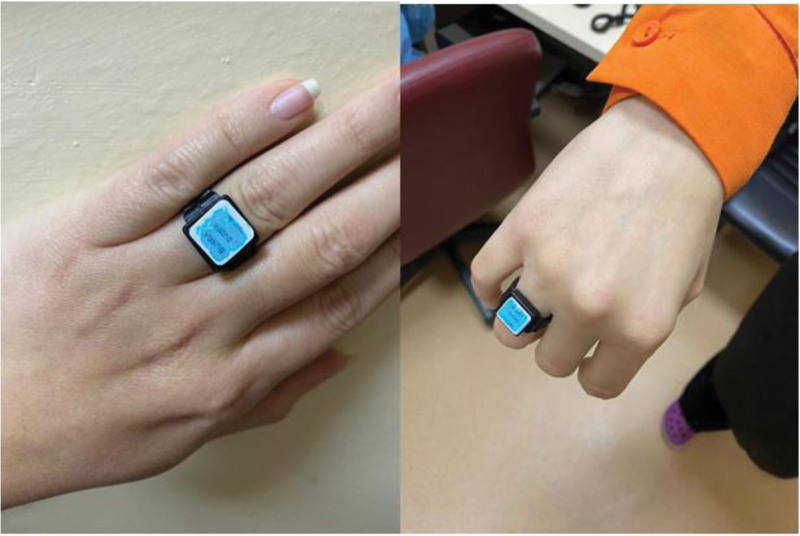
Position of the dosemeter.

This study is a retrospective cross-sectional study for 9 years (2013–2021) conducted in PET/CT units in 5 Turkish hospitals. All hospitals perform F-18 FDG PET/CT examinations. A total of 502 radiation dose records were retrieved from the radiation dosimetry service, and 113 cumulative extremities equivalent doses (HP0.7) for 55 medical workers working in PET/CT were included in the study. Workers included in the study were radiographers/radiologic technologists, physicists, and nurses. All workers are instructed to wear a dosimeter at the finger base to measure the extremities dose (Fig. 1). The radiation dosimeter used by workers was an optically stimulated luminescence (OSL) and Thermoluminescent dosimeter (TLD) ring dosimeter. Hospitals 1, 2, and 5 used OSL, and Hospitals 3 and 4 used TLD (Table 1). All workers wore the dosemeter on hand. The OSL dosimeter is ideal for monitoring medical radiation workers as it is a sensitive dosimeter used to monitor the accumulated dose of X, gamma, beta, and neutron radiation. The employed OSL dosimeter had sensitivity = 0.1 mSv (up to 10 Sv), detection range = 25 keV—1.25 MeV, and working temperature = 0°C to 50°C. The validation method was used according to the TS-EN-62387 standard. TLDs are a common dosemeter that is lithium fluoride-based TLD-100 (Zeff = 8.14; ρ = 2.635 g/cm^3^).^[6]^ TLDs are used to measure occupational exposure dose, including the Hp(10), Hp(0.07), and extremity. It is calibrated using a uniform photon beam from a Cesium-137 source and has been read using an automated TLD reader along with Windows-based Radiation Evaluation and Management System (WinREMS) software.^[7]^

**Table 1:** Hospitals information.

	Hospital 1	Hospital 2	Hospital 3	Hospital 4	Hospital 5
Dosemeter	OSL	OSL	TLD	OSL	TLD
Machine	PHILIPS	GE	PHILIPS	Siemens	Siemens
Average cases per yr	3628	1280	6858	1007	4310
Type of injector	Manual	Manual	Manual	Manual	Manual
Average dose	29.84	6.19	24.93	1.98	26.36

OSL = optically stimulated luminescence, TLD = thermoluminescent dosimeter.

Statistical analysis. All data were grouped into categories based on occupation. Data were collected, categorized, and processed using the Statistical Package for Social Sciences (SPSS), software package version 25. The quantitative variables were expressed as mean ± Standard Deviation (SD). The current study is quality improvement research; therefore, it was exempted from ethical approval.

## 3. Results

**Figure 2.**
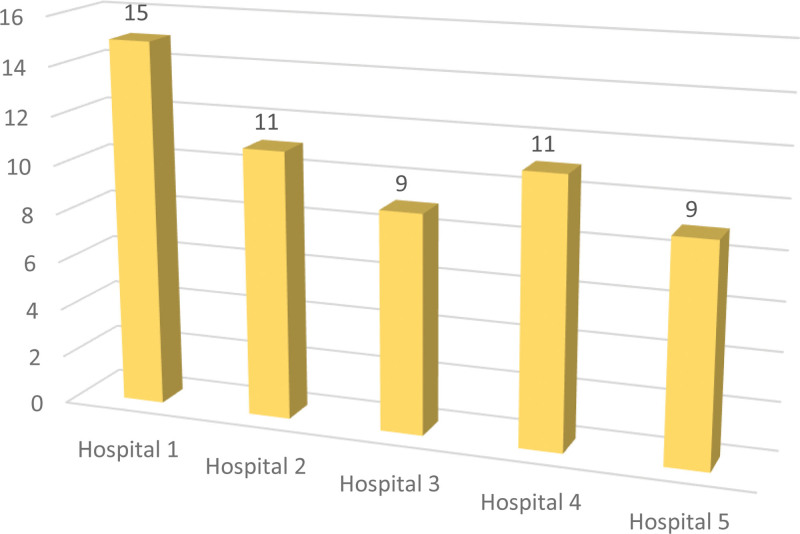
Number of workers per hospital.

A total of 502 readings were obtained throughout the study period from 55 workers employed across 5 hospitals. Nuclear physicists (3.6%, n = 2), nurses (9.1%, n = 5), and radiographers/radiologic technologists (87.3%, n = 48) working in the PET/CT department in different roles were included in the study. The number of workers per hospital is shown in Figure 2. The number of participants from 2013 to 2017 was approximately 6. From 2018 to 2021, the number of participants has reduced from 41 to 14 (Table 2). The staff utilized 2 types of dosimeters: OSL dosimeter (65.5%, n = 36) and TLD dosimeter (34.5%, 19).

**Table 2:** Average, min, max, SD for workers per yr.

Yr	N	Minimum	Maximum	Mean	Std. Deviation
2013	6	7.3	42.5	25.3	14.4
2014	5	6.0	69.0	30.6	27.4
2015	6	2.4	23.8	12.5	8.8
2016	6	1.2	25.5	10.4	10.7
2017	6	0.5	18.9	10.5	7.9
2018	41	0.3	84.9	9.5	14.4
2019	26	0.5	157.2	36.8	45.3
2020	23	0.4	78.3	21.5	26.0
2021	14	0.5	37.4	9.3	11.6

**Figure 3.**
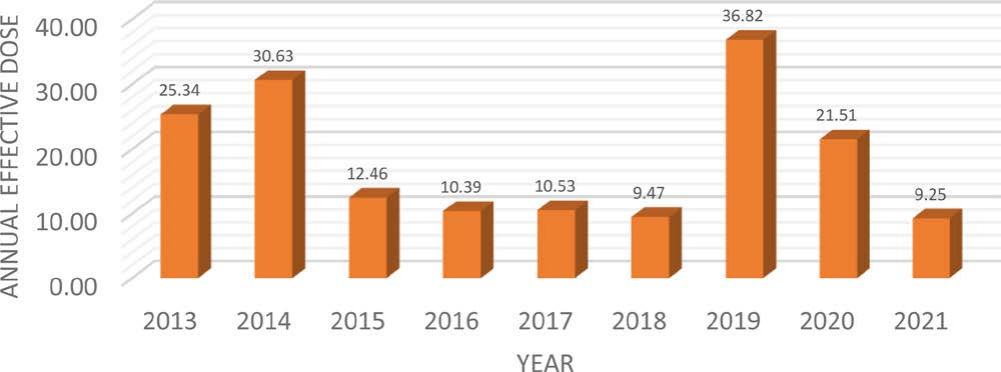
Annual effective dose in mSv. mSv = millisievert.

The average annual effective dose for all workers was 14.5 mSv (SD 17.73), ranging from 0.21–157.21 mSv. The yearly analysis showed that the average annual effective dose for all workers ranged from 9.24 mSv (2021) to 36.83 mSv (2019) throughout the study period (Fig. 3).

The average annual effective dose was calculated for each worker group (Table 3).

**Table 3:** Average, min, max, SD for workers group.

Worker	Minimum	Maximum	Mean	Std. Deviation
Nurse	0.32	65.58	15.19	19.47
Nuclear physicist	1.20	24.43	8.62	10.49
Radiology technician	0.42	157.21	14.71	18.03
All workers	0.32	157.21	14.5	17.73

It was revealed that the nurse received the highest average annual effective dose of 15.19 mSv (SD 19.46). The yearly analysis showed that the maximum dose of 157.21 mSv was received by a radiographer based in Hospital 1 in 2019. The average dose per hospital was 29.84, 6.19, 24.93, 1.98 and 26.36 for hospitals 1, 2, 3, 4 and 5, respectively (Table 4).

**Table 4:** Average, min, max and SD for hospital.

Hospital	Minimum	Maximum	Mean	Std. Deviation
All Hospital	157.21 (Rad Tech)	0.21 (Rad Tech)	18.88	27.06
Hospital 1	157.21 (Rad Tech)	0.54 (Rad Tech)	29.84	33.03
Hospital 2	12.20 (Rad Tech)	0.23 (Rad Tech)	6.19	4.32
Hospital 3	78.32 (Rad Tech)	0.99 (Rad Tech)	24.93	25.38
Hospital 4	8.14 (Rad Tech)	0.21 (Rad Tech)	1.98	2.09
Hospital 5	84.99 (Rad Tech)	0.42 (Rad Tech)	26.36	28.72

Rad Tech = Radiography Technologist.

A t test (*P* < .05) was conducted to analyze the significant difference in the average annual effective dose among the nurse and radiographers/radiologic technologists; there was no statistically significant difference (*P* = .955).

## 4. Discussion

The number of workers increased dramatically in 2018 and decreased by half in 2019 and the years onward. The number of PET/ CT scans declined after COVID-19^[8]^ as many hospitals all over the world,^[9,10]^ including Turkey,^[11]^ directed their workforce towards x-ray and CT scans to accommodate the staff shortage and the need to diagnose CVOID-19.

The average annual effective extremities dose for all workers was 14.5 mSv (SD 17.73). Also, the yearly analysis showed that the average annual effective dose for all workers ranged from 9.24 mSv (2021) to 36.83 mSv (2019) throughout the study period (Fig. 2). In the current study, the overall extremity occupational radiation exposure was well below the annual dose limit of 500 mSv.^[5]^ Nevertheless, the Extremity occupational exposure was higher than previously published results by Pavičar et al^[3]^ and Adliene et al^[12]^ but it is lower than the results shown by Kaljevic et al^[13]^ and other studies (Table 5).

**Table 5:** Comparison between current results and other studies available in the literature.

	All workers	Technologist	Nurse	Nuclear physicist
Current study	14.5mSv	14.71 mSv	15.19 mSv	8.62 mSv
Pavičar et al^[3]^	-	4.37 μSv	6.55 μSv	-
Saad et al^[14]^	-	38.77 μSv	59.6 μSv	203 μSv
Khouqeer^[15]^	223 mSv	-	-	-
Kaljevic et al^[13]^	266 mSv	-	-	-
Wrzesien et al^[16]^	>500 mSv	-	-	-

mSv = millisievert.

However, there was no statistically significant difference in workers’ average annual effective dose. Nurses and radiographers/radiologic technologists had higher average annual effective doses of 15.19 mSv and 14.71 mSv. Also, the yearly analysis showed that the maximum dose of 157.21 mSv was received by a radiographer/radiologic technologist. It has been reported that radiographers/radiologic technologists working in PET/CT have higher absorbed radiation doses compared to other workers.^[17]^ All hospitals are equipped with manual injectors, and studies showed that the radiation exposure to hands showed increment for staff using semi-automated or manual injecting systems.^[17,18]^

Protection against radiation necessitates the effective implementation of knowledge. Hence, developing a culture of radiation protection can significantly decrease the amount of radiation dose received by staff members. In order to close the gap between theory and practice, continuous review and ongoing improvement are required.^[19]^ Increased knowledge and awareness of the necessity to follow radiation protection requirements are necessary.^[20]^ Training programs play an essential role in reinforcing radiation protection measures to improve the practice. A previous study recommended tailored radiation protection training for nurses.^[21]^ The literature argues for the consequences of the experience on occupational radiation exposure. Workers with limited experience receive significantly higher doses to the fingers than highly skilled.^[22,23]^ Nevertheless, proper practice of radiation protection and correctly using equipment and tools are more important than experience.^[24]^ Personalized feedback on occupational doses can be delivered to medical professionals, and discussion of pitfalls can reduce occupational radiation exposure.^[25]^

Based on inverse law, the amount of radiation that is absorbed is inversely related to the square of the distance to the radiation source. The dose received by the fingertips cannot be represented by ring dosimeters, which are worn on the basis of the fingers. Thus, research suggests that the radiation dose recorded at the bases of the fingers may be 40 times higher than the amount measured at the thumb and index finger fingertips.^[17]^ Therefore, the exposure at the fingertips might be very high compared to ring dosimeter readings. Therefore, applying sufficient shielding when administering radiopharmaceuticals is essential to minimize radiation exposure to the extremity. However, the use of semi- and fully automatic dispensers and injectors has been found to decrease the dose to extremities.^[26]^

## 5. Conclusion

This study calculated the annual extremity occupational doses for PET/CT workers in 5 hospitals. PET/CT workers are divided into groups based on their occupation, including nurses, radiographers/radiologic technologists, and nuclear medicine physicists. For all occupations, the extremity dose was less than the annual dose limit (500 mSv), but the highest dose (157.21) was received by a radiographer/radiologic technologist, and nurses received the highest average annual effective dose (15.2 ± 19.46). The maximum dose received by a radiography technologist. The nuclear medicine physicist had the lowest dose average annual effective dose (8.6 ± 10.5). The result shows a wide variation in dose among the workers, underlining a need for careful assessment of working conditions to ensure safe practice for all workers.

## 6. Limitations

The limited number of monitored nuclear medicine physicists is a limitation of the study. In addition, the practice of radiation protection, use of appropriate radiation protection tools and compliance with optimal dose monitoring were not considered in this analysis. Future studies concentrating on assessing compliance with radiation dose monitoring and practice of radiation protection measure are recommended. Also, the results cannot be generalized for PET/CT workers in other hospital. Therefore, each hospital can assess the occupational radiation dose for radiology workers in their department.

## Author contributions

**Conceptualization:** Rabiye Uslu Erdemir, Baris Cavli, Huseyin Ozan Tekin.

**Formal analysis:** Mohamed Mahmoud Abuzaid, Wiam Elshami.

**Investigation:** Wiam Elshami.

**Methodology:** Wiam Elshami.

**Validation:** Rabiye Uslu Erdemir, Huseyin Ozan Tekin.

**Writing – original draft:** Mohamed Mahmoud Abuzaid, Wiam Elshami.

**Writing – review & editing:** Rabiye Uslu Erdemir, Mohamed Mahmoud Abuzaid, Baris Cavli, Huseyin Ozan Tekin, Wiam Elshami.
